# Territoriality and Conflict Avoidance Explain Asociality (Solitariness) of the Endosymbiotic Pea Crab *Tunicotheres moseri*

**DOI:** 10.1371/journal.pone.0148285

**Published:** 2016-02-24

**Authors:** Louis J. Ambrosio, J. Antonio Baeza

**Affiliations:** 1 Department of Biological Sciences, Clemson University, Clemson, South Carolina, United States of America; 2 Smithsonian Marine Station at Fort Pierce, Fort Pierce, Florida, United States of America; 3 Departamento de Biología Marina, Facultad de Ciencias del Mar, Universidad Católica del Norte, Larrondo, Coquimbo, Chile; Biodiversity Research Center, Academia Sinica, TAIWAN

## Abstract

Host monopolization theory predicts symbiotic organisms inhabiting morphologically simple, relatively small and scarce hosts to live solitarily as a result of territorial behaviors. We tested this prediction with *Tunicotheres moseri*, an endosymbiotic crab dwelling in the atrial chamber of the morphologically simple, small, and relatively scarce ascidian *Styela plicata*. As predicted, natural populations of *T*. *moseri* inhabit ascidian hosts solitarily with greater frequency than expected by chance alone. Furthermore, laboratory experiments demonstrated that intruder crabs take significantly longer to colonize previously infected compared to uninfected hosts, indicating as expected, that resident crabs exhibit monopolization behaviors. While territoriality does occur, agonistic behaviors employed by *T*. *moseri* do not mirror the overt behaviors commonly reported for other territorial crustaceans. Documented double and triple cohabitations in the field coupled with laboratory observations demonstrating the almost invariable success of intruder crabs colonizing occupied hosts, suggest that territoriality is ineffective in completely explaining the solitary social habit of this species. Additional experiments showed that *T*. *moseri* juveniles and adults, when searching for ascidians use chemical cues to avoid hosts occupied by conspecifics. This conspecific avoidance behavior reported herein is a novel strategy most likely employed to preemptively resolve costly territorial conflicts. In general, this study supports predictions central to host monopolization theory, but also implies that alternative behavioral strategies (i.e., conflict avoidance) may be more important than originally thought in explaining the host use pattern of symbiotic organisms.

## Introduction

Territoriality (herein defined as any behavior employed by an individual to defend a resource from intrusion by another individual) is a widespread behavioral strategy in terrestrial and aquatic organisms [1–3]. Theory predicts territoriality to be adaptive only when economically viable; when the net benefits from defending a resource are greater than the cost associated with its defense [4–6]. Benefits associated with territorial behaviors include but are not limited to increased access to mates [6–7], food [8], and refuges [9–10]. Conversely, the costs associated with territorial behavior include, among others, energy expended in defense [11], injuries sustained while defending resources [12–13], and/or mortality [13–14].

A variety of environmental conditions can alter the costs and benefits of defending a shelter, and thus, are expected to influence the expression of territoriality. Overall, territorial behavior is predicted to evolve when resources are highly aggregated and/or scarce, and when intrusions by competitors are infrequent, given that the conditions above decrease the costs of guarding [5]. The influence of environmental conditions on the expression of territoriality has been explored in a variety of vertebrate taxa [5, 15–16]. However, relatively few studies have explored the expression of territoriality and the conditions favoring it in terrestrial and aquatic invertebrates.

Among invertebrates, symbiotic crustaceans represent a good model to study the effect of environmental conditions on the expression of territoriality. Symbiotic crustaceans inhabit hosts with diverse ecologies and exhibit a wide spectrum of host-use patterns. For instance, some symbiotic crustaceans inhabit hosts in large well structured kin societies (e.g., various alpheid shrimp from the genus *Synalpheus*: [17]), others cohabit in or on their hosts as heterosexual pairs [12, 18–20], and even others are almost invariably found living solitarily within or on host individuals [21–24]. The various host-use patterns above likely reflect differential host-resource defense strategies by symbiotic crustaceans that are expected to be driven by host intrinsic and/or extrinsic traits (i.e. host morphology, structural complexity, abundance). Furthermore, host resources are a valuable refuge for symbiotic crustaceans because they often provide a direct or indirect food source [25], mating arena [7], and protection from adverse biotic and abiotic conditions (e.g., predation [26]; desiccation [27]).

In symbiotic crustaceans, host characteristics such as abundance, body size (relative to that of the symbiotic guest crustacean), and morphological complexity have been proposed to be the most relevant factors modulating the economics of territoriality (host monopolization theory: [3, 28–30]). Relatively small and morphologically simple hosts are thought to be economically defendable because the benefits resulting from host monopolization (i.e., shelter against abiotic and biotic conditions) outweigh the (relatively low) cost of defense. In turn, in large and structurally complex hosts, the costs of defense (energy expenditure) outweigh the benefits of host defense [3]. Overall, symbiotic crustaceans inhabiting relatively small and simple host refuges should exhibit a solitary or socially monogamous lifestyle and display territoriality while those inhabiting large and structurally complex hosts should not display resource monopolization behaviors and live in larger aggregations [3]. Studies conducted during the last few decades have supported the notion above [20, 31–32]. For instance, Baeza and Thiel [3] demonstrated that structurally simple and small hosts (e.g., sea anemones) harbor ectosymbiotic crab species that live solitarily and display territorial behavior while structurally complex and large hosts (i.e., sea urchins) harbor ectosymbiotic crabs that live in aggregations and do not exhibit territorial behavior. Still, only a few studies to date have clearly demonstrated the effect of host traits on the expression of territorial behavior using field data and experimental trials [23, 33].

**Fig 1:**
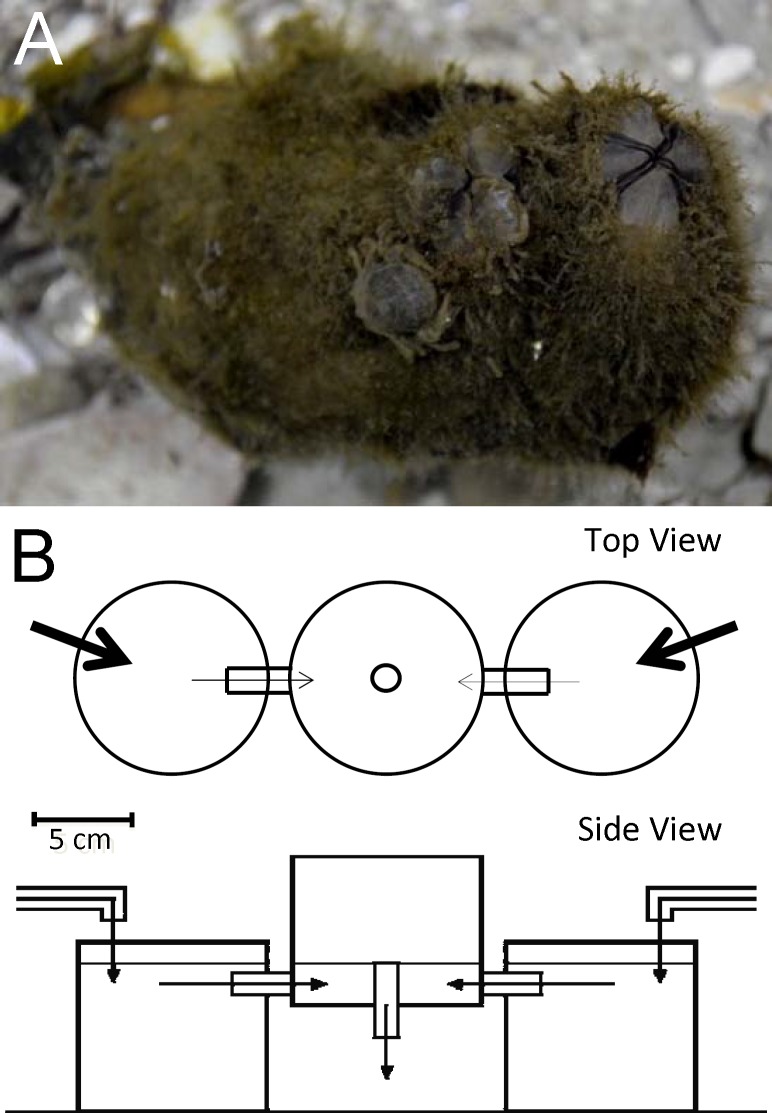
Symbiotic associates *Tunicotheres moseri* and *Styela plicata*; chemoreception test apparatus. (a) Symbiotic crabs *Tunicotheres moseri* on the tunic and entering the atrial siphon of host ascidian *Styela plicata*. Crab on the tunic measures 7mm carapace width. (b) Top view (above) and side view (below) of the chemoreception test chamber used to test juvenile crab preference for occupied and vacant hosts.

The present study tests the prediction that symbiotic crustaceans inhabiting small and morphologically simple hosts should exhibit territoriality ([3] and references therein). We used the endosymbiotic pea crab *Tunicotheres moseri* (Fig 1) and its morphologically simple and small ascidian host *Styela plicata* (Fig 1) as a model system to test this prediction [34–35]. This monotypic crab is considered to be a commensal symbiont and typically inhabits the atrial chamber of a small number of solitary ascidian species (e.g., *S*. *plicata*, *Phallusia nigra*, *Molgula occidentalis*, and *Polycarpa spongiabilis*) in the Caribbean and eastern Gulf of Mexico [24, 34–35 and references therein]. The atrial chamber (a subdivision of the already restrictive internal body cavity) of ascidians is a relatively small and structurally simple refuge that likely protects *T*. *moseri* from potential predators such as fishes (e.g., pinfish *Lagodon rhomboides*, grey snapper *Lutjanus griseus*) known to prey upon small crustaceans such as *T*. *moseri* [36–37]. In addition, ascidians most likely provide a food source, a mating arena, and a nursery ground for pea crabs [35]. The host characteristics and the high value that ascidians represent suggest that *T*. *moseri* should exhibit territorial behavior. In partial agreement with this expectation, a recent study reported that *T*. *moseri* inhabits ascidians solitarily but only during a portion of the year (e.g., during fall and spring but not during summer [24]). Additionally, the same authors also suggest that females rather than males form long term associations with hosts potentially indicating a female specific bias for territoriality since that sex may demonstrate greater host fidelity [24]. Temporal or spatial variations in host availability (abundance) might explain the shifts in host-use reported for *T*. *moseri* [29–30]. Importantly, Hernández et al. [24] did not experimentally test if the solitary habit of *T*. *moseri* during part of the year was the product of territoriality. Indeed, concomitantly or alternatively to territoriality, avoidance of already occupied hosts by crabs might represent a mechanism that can drive the solitary habit reported for this symbiotic crab [24]. *Tunicotheres moseri* adults have been reported to sense and use waterborne chemical cues derived from hosts and conspecifics to locate hosts and to acquire mating partners [34]. *Tunicotheres moseri* may also employ chemical cues to avoid colonization of already occupied hosts, and this behavior might determine the solitary habit of this crab.

If *T*. *moseri* is a territorial species, we expect that (1) *T*. *moseri* will display a solitary habit in hosts as predicted by host monopolization theory and (2) crabs will engage in overt agonistic behaviors when competing for ascidians. Instead, if the solitary habit of *T*. *moseri* is due to avoidance of already occupied hosts, we expect that (3) searching crabs will chemically sense conspecific competitors and use this information to choose unoccupied hosts in an attempt to reduce conflict over host individuals. Henceforth, the active avoidance of occupied hosts and preference for vacant hosts will be referred to as ‘conflict avoidance’.

## Materials and Methods

### Study sites and collection of *Tunicotheres moseri*

We described the population dynamics and host use pattern of *T*. *moseri* in its common host, the ascidian *S*. *plicata*, in Tampa Bay estuary (N 27° 35’12.6”, W 82° 37’06.7”), south west Florida, USA. For this purpose, ~40 ascidians were haphazardly collected from artificial concrete jetties located near the Sunshine Skyway Bridge at the mouth of the bay while free diving during the first week of each month from May, 2004 to April, 2005. Each collected ascidian was placed in a 207 ml Whirl Pak® plastic bag as soon as it was dislodged from the substrate (while still underwater) and transported within an ice-chest to the University of Tampa Marine Laboratory for further processing. In the laboratory, the total length (TL) of each ascidian was measured from the base of the tunic to the incurrent siphon using a manual caliper (precision = 0.1 cm). Ascidians were then gently dissected with a scalpel to determine the presence or absence of *T*. *moseri* and to record the total number of crabs per host individual. Next, the carapace width (CW) and sex of each crab was estimated. CW was measured at the widest section of the carapace with a manual caliper to the nearest 1 mm. Sex was determined through examination of shape and width of the abdomen. Males display a narrow abdomen while females exhibit a broad abdomen covering the entire ventral side of the body [24]. Individuals with indistinguishable sex using the metric above were considered juveniles.

Little is known about the seasonal abundance of *S*. *plicata* or the influence of host availability on *T*. *moseri* population distribution. Thus, we measured *S*. *plicata* seasonal density and size frequency distribution in 0.5 cm size class intervals at the Sunshine Skyway Bridge study site. Host density data were recorded by counting the number of ascidians within a quadrant (1 m^2^) placed on the concrete substrate during each sampling event.

Lastly, uninfected ascidians used in experiments were collected from floating docks located at the University of Tampa Marine Science Field Station between May and September 2007 and between October and December 2014. This latter location was chosen because it lies outside of the discrete range of *T*. *moseri*. All collected *S*. *plicata* and *T*. *moseri* (removed or not from their ascidian hosts) were maintained under laboratory conditions (Salinity: 32 ppt, Temperature: 22–23°C, photoperiod: 13.5 h light and 10.5 h dark) before being used for experiments.

All collection sites and organisms used during this study fell under the management of the Florida Fish and Wildlife Conservation Commission and did not require specific collection permits other than a recreational fishing license which was provided by LJ Ambrosio. In addition, the species used in this study were managed as unregulated species by both the state of Florida and United States Fish and Wildlife Services and did not require the implementation of any specific animal use protocols according to the Institutional Animal Care and Use Committee.

### Population distribution of *Tunicotheres moseri*

Prevalence and population distribution of *T*. *moseri* in its host *S*. *plicata* were recorded on a per season basis to document possible temporal changes in these population parameters. We examined whether or not *T*. *moseri* lives solitarily, in aggregations, or in pairs in its host *S*. *plicata*. An expected random Poisson distribution was compared to the observed population distribution (i.e., the frequency of occurrence of hosts without crabs and with different numbers of crabs) [38]. Significant differences between observed and expected distributions were determined using a Chi-square test of goodness-of-fit. If the test above detected significant differences between the compared distributions, we employed an a-posteriori 'Subdivision Chi-square test' to detect significant differences between specific frequencies (e.g., observed versus expected frequency of solitary crabs). If *T*. *moseri* is a territorial species, as predicted by host monopolization theory [3], we expected that this species would display a uniform distribution (crabs living solitarily) in *S*. *plicata*.

### Testing for territoriality in *Tunicotheres moseri*

We designed two behavioral experiments to determine the role of agonistic interactions between conspecifics in explaining the solitary lifestyle (see results) of *T*. *moseri*.

#### Experiment 1: Territoriality between two intruder adult crabs

In the first experiment, we tested for the expression of overt agonistic behaviors in adults of *T*. *moseri*, by simultaneously exposing two adult female crabs of unequal CW (> 2 mm difference) to a single unoccupied ascidian of *S*. *plicata*. We specifically used adult female crabs in this and subsequent experiments (see below) given that females are considered to be sedentary (compared to males) and establish long-term associations with ascidian hosts [24]. Crabs ranged in size from 5–9.5 mm CW. To distinguish the crabs during the experiment, the carapace of the smaller and larger female was tagged with a yellow and red dot (diameter = 1 mm), respectively. In each of a total of 40 replicates, the two crabs together with the unoccupied ascidian were placed in one compartment of a multi-chambered perforated clear polypropylene box (10.16 cm height x 7.62 cm wide). The length of the compartment was adjusted to accommodate crabs and ascidians of different sizes during the experiment. Experimental boxes were placed in water tables with circulating laboratory-conditioned seawater (Salinity: 32 ppt, Temperature: 22–23°C, Photoperiod: 13.5 h light and 10.5 h dark).

At the start of each replicate, both experimental crabs were placed side-by-side 7.62 cm away from the uninfected ascidian located at the center of each compartment. In each replicate, we recorded the first crab (i.e., smaller or larger) to enter the host individual through the atrial chamber and any agonistic display between crabs when attempting to infect the offered ascidian host during a 24 h period. Crab behaviors recorded included (1) fighting (i.e., physical contact between the chelipeds and/or pereopods of the two experimental crabs), (2) signaling (i.e. crabs raising the body above the substrate, lateral extension of the chelipeds [12, 39]) and (3) escape responses (crabs retreating after fighting). The importance of body size in determining the winner of agonistic interactions when conspecifics compete for resources is well established in crustaceans, including crabs [12, 40–43]. Thus, we expected the larger of the two experimental crabs to invade the host first during our experiment. We used a Chi-square test of goodness-of-fit to compare the frequency with which large and small crabs infected first the host individual to that predicted by a random binomial distribution [44].

#### Experiment 2: Territoriality between one resident and one intruder adult crab

**Fig 2:**
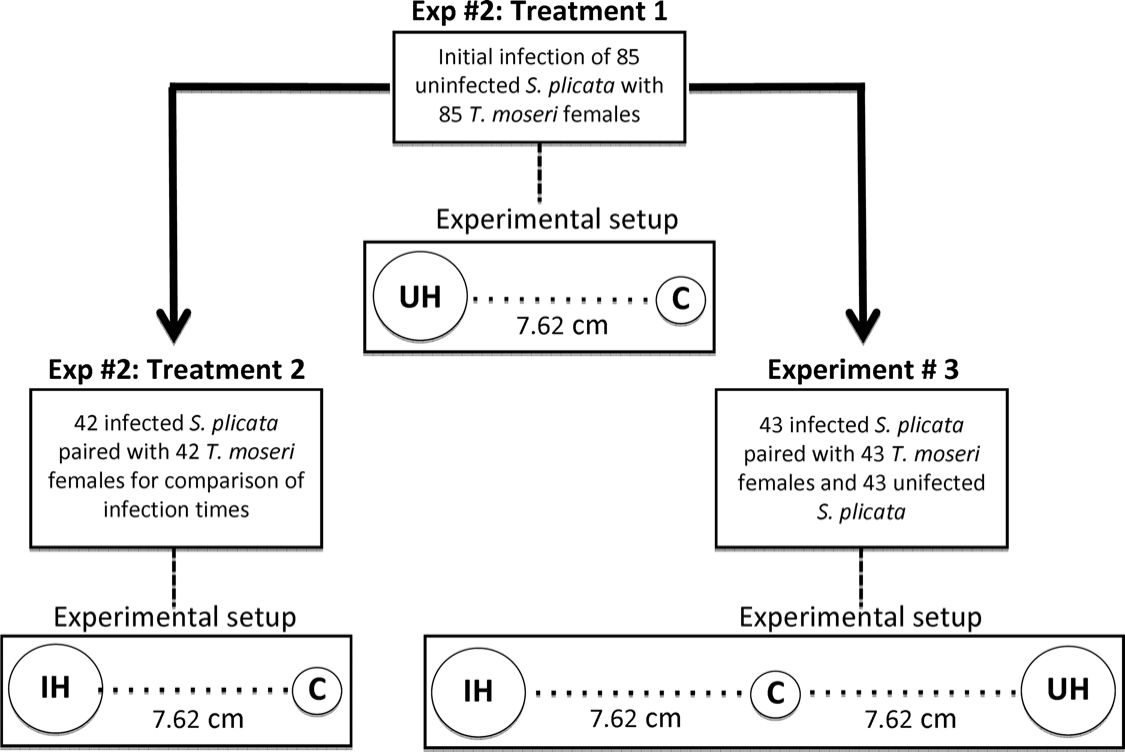
Flowchart of Experiments 2 and 3. Flowchart depicting the experimental design and setup of Experiments 2 and 3. Infected hosts generated in Experiment 2, Treatment 1 were used in Experiment 2, Treatment 2 and Experiment 3. Abbreviations UH, IH and C stand for uninfected host, infected host and crab, respectively.

In the second experiment, we tested for territoriality in *T*. *moseri* using an 'intruder' crab that was offered either an uninfected (treatment 1) or already occupied (treatment 2) individual of *S*. *plicata* (Fig 2) and recorded every 4 h (beginning 2 h after the start of the experiment for a total period of either 16 h or 24 h in treatment 1 and 2, respectively) the moment during which each focal crab (i.e. the crab making the choice) [i] started entering the ascidian through its oral siphon (time to initiate host infection) and [ii] completed host infection entering the atrial chamber of the ascidian host (and thus, crabs were not visible anymore in the experimental chambers). The maximum period of data collection was increased in treatment 2 from 16 to 24 h anticipating that resident crabs will likely delay host colonization by intruder crabs. We conducted a total of 85 replicates in treatment 1 and 42 replicates in treatment 2. Infected ascidians used in treatment 2 were those hosts infected by crabs during treatment 1. The size of each focal female crab (CW) and ascidian (TL) were measured before the start of each replicate in each treatment. The 42 replicates of treatment 2 were additionally divided into two different size-specific resident and intruder crab pairings. Twenty-one of the 42 replicates had a larger resident and a smaller intruder crab, whereas the remaining replicates (n = 21) had a smaller resident and a larger intruder crab allowing us to test for the effect of body size on contest outcome. The size disparity between resident and intruder crabs was not less than 2 millimeters.

In each replicate of this experiment, we used the same test chambers, water tables, and laboratory conditions described above (Experiment 1) and the ascidians offered for colonization to intruder crabs, either infected or not, were placed 7.62 cm apart from the intruder crab at the start of each replicate (Fig 2). All ascidians in treatment two were dissected after 24 h and the location of each experimental crab (resident and intruder) within the atrial chamber of each ascidian host was recorded.

We compared the time to infection initiation and completion between the two treatments using the Wald Chi-square method, part of Cox’s maximum likelihood regression [45] and an event time (survival) analysis technique, in the statistical program JMP 12 [46]. We expected resident crabs to hinder or rebuff an intruder’s attempt to enter the host. Therefore, if territorial behavior explains the solitary habit of *T*. *moseri*, we expected that intruder crabs would take longer to initiate and complete the infection in treatment 2 than in treatment 1. We also expected fewer intruder crabs to infect a host within the experimental period in treatment 2 compared to treatment 1. Comparisons between the proportions of crabs successfully completing infections and the proportion of smaller and larger intruder crabs successfully completing infections were analyzed using Fisher’s exact test [47].

### Testing for conflict avoidance in *Tunicotheres moseri*

#### Experiment 3: The effect of host infestation on host choice by adult crabs

We offered adult female crabs a choice between infected and uninfected ascidians to determine whether or not the presence of conspecifics alter their host preference. During this experiment, females were exposed to both chemical and tactile stimuli given that adult females have been shown in a previous study to use a combination of chemical and tactile cues to recognize conspecifics and hosts [34]. In each replicate, we placed an infected ascidian (harboring an adult female crab) with a second uninfected ascidian matched in body size (total length within ± 0.5 cm) 16 cm apart from one another in a compartment. Next, a second 'focal' adult female of *T*. *moseri* (between 5 and 8 mm CW) was placed equidistantly (7.62 cm) between the two ascidians (Fig 2). The preference of this 'focal' crab for infected vs uninfected ascidians was recorded after a 24 h experimental period when the two ascidians were dissected and the presence/absence of the 'focal' crab was recorded. Crabs were recorded as choosing an ascidian only if found within its atrial chamber or at the exhalant siphon (in the process of entering the ascidian) at the end of the experimental period. We used a Chi-square test of goodness-of-fit to compare the frequency of crabs choosing between infected and uninfected ascidians to that predicted by a random binomial (1:1) distribution [44]. If conflict avoidance was the mechanism driving the solitary habit of *T*. *moseri*, we expected focal crabs to choose uninfected over infected hosts. Infected ascidians used in this experiment were a total of 43 out of the 85 originally infected in treatment 1 of Experiment 2 and not used in treatment 2 of the same experiment.

#### Experiment 4: The effect of host infestation on host choice by juvenile crabs

Although both chemical and tactile cues were presented for host discrimination in the adult conflict avoidance experiment above, we offered only chemical cues when testing for juvenile conflict avoidance. Justification for this lies in the fact that *T*. *moseri* is known to use chemical stimuli alone as a means of host and conspecific recognition [34] and that chemoreception is commonly used in other invertebrates as a means of habitat selection during settlement or early in life [48–50].

We tested the preference for cues derived from infected and uninfected hosts in a PVC, three-chambered choice apparatus (Fig 1). The choice apparatus used in this study consisted of a central chamber in which test subjects were positioned at the start of each replicate of this experiment and two satellite chambers that were connected to the central chamber and received water from experimental treatments (Fig 1). All early juvenile crabs used in this experiment were collected within 12 h of release from the brood pouch of seven different females and replicates (see below for details) were conducted under constant total darkness to eliminate any effect of visual cues on host choice. All experiments used seawater that was aged for 7 days in 19 l carboys to allow for the degradation of latent chemical stimuli and then aerated 24 h prior to use in this experiment, hereafter referred to as laboratory conditioned water. Treated water was made by holding 10 *S*. *plicata*, either uninfected or each infected with a pair of conspecific females, in 8 l of aerated laboratory conditioned water for 24 h. The satellite chamber receiving treatment water was selected randomly for each trial and the choice apparatus was rinsed in hot tap water and then control water between uses. In all experiments, water flow of 10 ml/min dripped into each satellite chamber and drained through a central standpipe in the center chamber. Only one crab was tested at a time to eliminate the influence of conspecific interactions that might bias the behavioral response to test stimuli. The same test chambers used in this experiment were tested and confirmed unbiased, regarding water flow dynamics and test subject behavioral influence, in a previous study [34].

To test the impact of conspecifics on host preference by early juvenile crabs, we first filled the choice apparatus with laboratory conditioned water and gently placed a recently released *T*. *moseri* instar in the center of the central chamber with a 3 ml plastic pipette. A constant flow of control water was delivered to each satellite chamber and circulated through the apparatus for a 30 min acclimation period. After this initial acclimation period, uninfected and infected treatment water was introduced into opposite satellite chambers. Initially, crab location at intervals of 1 h, 2 h, 3 h, and 4 h were observed to confirm that crabs did not change location once they had relocated to one of the satellite chambers. Results from these initial observations indicated that once a choice was made, crabs did not switch between chambers. Thus, behavioral responses were recorded after a 4 h period as: 1) preference for uninfected host if the instar relocated to the satellite chamber corresponding to uninfected host stimuli or 2) preference for infected host if the instar relocated to the satellite chamber corresponding to infected host stimuli for a total of 50 replicates. We used a Chi-square test of goodness-of-fit to compare the frequency of early juveniles choosing between infected and uninfected hosts to that predicted by a random binomial (1:1) distribution [44]. If conflict avoidance was a relevant mechanism explaining the solitary habit of this crab, then, we expected juvenile crabs to choose uninfected over infected hosts.

## Results

### *Styela plicata* population and prevalence of *Tunicotheres moseri*

The density of the ascidian *S*. *plicata* at our study site ranged from 3 to 10 individuals m^-2^. The greatest average host density occurred in fall with 9.33 ± 0.57 individuals m^-2^, while the lowest average host density was observed during winter (3.66 ± 0.28 individuals m^-2^). Over the entire study period, *S*. *plicata* ranged in body size from 3.5 cm to 10.0 cm TL with an average TL of 6.18. Seasonally, the average body size (±SE) of ascidians ranged from 6.57 ± 0.11cm in fall to 5.69 ±0.04 cm in spring.

**Fig 3:**
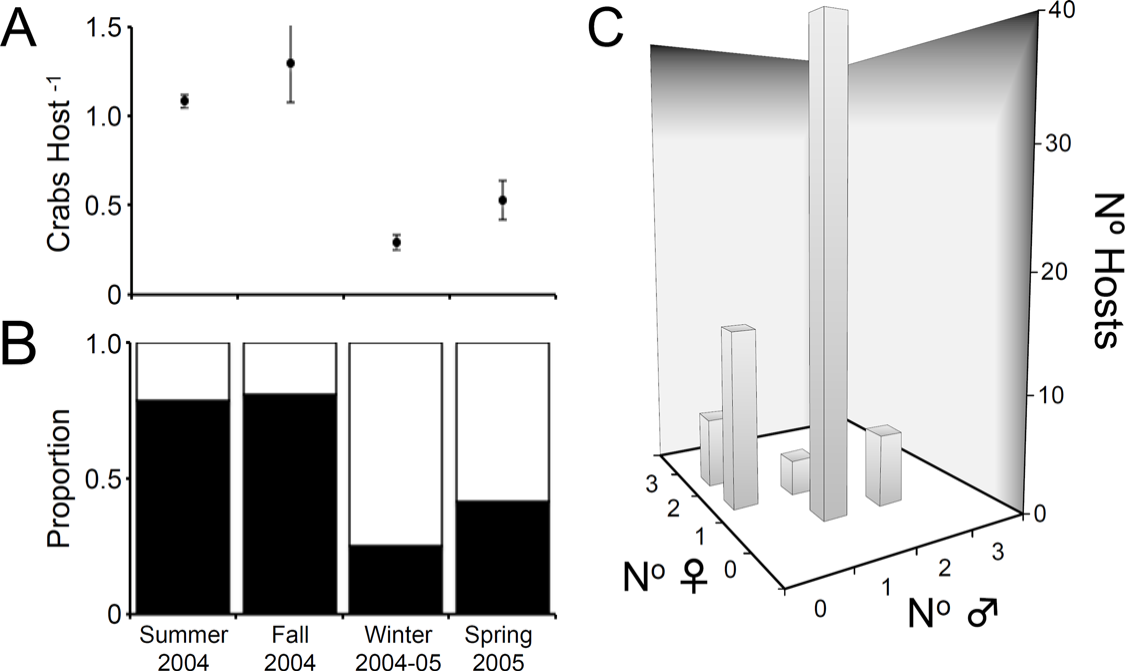
*Tunicotheres moseri* density, prevalence, sex distribution and group size in *Styela plicata*. (a) Mean abundance (N° crabs hosts^-1^) of *Tunicotheres moseri* in the ascidian *Styela plicata* in Tampa Bay. (b) Prevalence of *T*. *moseri* in the ascidian *S*. *plicata* in Tampa Bay. Black bars represent proportion of ascidians infected with *T*. *moseri*, while white bars represent the proportion of uninfected ascidians collected. (c) Sex distribution of *Tunicotheres moseri* in hosts *S*. *plicata* harboring crab groups of different sizes.

A total number of 387 individuals of *T*. *moseri* were retrieved from 482 ascidians collected during the entire study period. Prevalence (reported as N° infected hosts divided by total N° examined hosts) of *T*. *moseri* in the ascidian *S*. *plicata* varied between 25% in winter and 81% in fall (Fig 3). An analysis of ascidians by size class (0.5 cm, range = 3.5–10 cm, data pooled altogether) revealed an increase in prevalence of *T*. *moseri* with increases in host body size. Large ascidians (>7.50 cm TL) had infection rates above 75% while the smallest host individuals (<4.0 cm TL) hosted symbiotic crabs only 40% of the time.

### Host use pattern of *Tunicotheres moseri*

**Fig 4:**
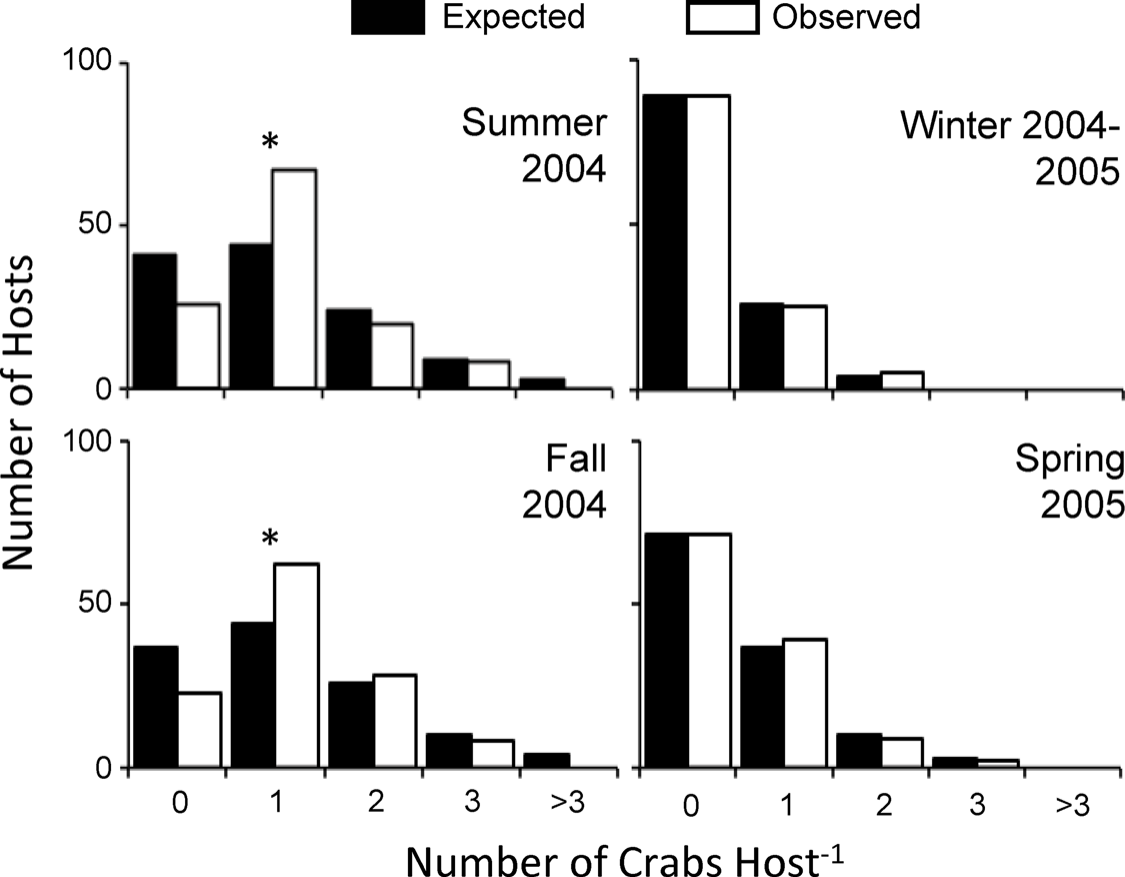
Seasonal population distribution of *Tunicotheres moseri* in *Styela plicata*. Population distribution of *Tunicotheres moseri* in the ascidian host *Styela plicata* during different seasons of the years 2004–2005. Observed frequency of occurrence of crabs in *S*. *plicata* differs significantly from the expected Poisson random distribution during summer and fall. Asterisks denote significant differences between the observed and expected frequency of occurrence of solitary crabs in ascidian hosts as indicated by *a posteriori* Chi-square tests of goodness of fit.

The mean abundance (reported as total N° crabs recovered divided by total N° hosts examined) of *T*. *moseri* in *S*. *plicata* fluctuated between 1.30 ± 0.22 (mean ± SE) crabs host^-1^ in fall and 0.29 ± 0.04 crabs host^-1^ in spring (Fig 3). The distribution of *T*. *moseri* in *S*. *plicata* was not random during summer and fall (observer vs. expected Poisson distribution, Chi-square test of goodness-of-fit; summer: *X*^*2*^ = 19.51, df = 3, *P* = 0.0002; fall: *X*^*2*^ = 15.38, df = 3, *P* = 0.0015) (Fig 4). The departures between observed and expected distributions during the above seasons were explained because single crab infections were observed with a frequency greater than expected by chance alone (summer: *X*^*2*^ = 18.89, df = 1, *P* = <0.0001; fall: *X*^*2*^ = 11.57, df = 1, *P* = 0.007). The frequencies of ascidians harboring solitary crabs during spring and winter were also greater than that expected by chance alone (Fig 4). Nonetheless, the population distribution of *T*. *moseri* did not differ statistically from a random Poisson distribution during the latter seasons (spring: *X*^*2*^ = 0.41, df = 2, *P* = 0.8123; winter: *X*^*2*^ = 0.28, df = 2, *P* = 0.8657).

### Group size and structure in *Tunicotheres moseri*

Due to the low frequency of crabs occurring in groups over the entire study period, we pooled the data from all seasons to examine group characteristics in *T*. *moseri*. A total of 193 ascidians harbored a single symbiotic crab over the entire study (40% of the total ascidians collected and 71% of the total ascidians infected). A total of 185 (96%) ascidians infected with a single symbiont harbored a female while only 4% of the host individuals with solitary crab infections harbored either a male or a juvenile (n = 8: four solitary males, and four solitary juveniles). Of the 58 ascidians harboring two crabs of defined sex, the number of heterosexual pairs present (n = 43) was significantly higher than the number predicted by a random binomial distribution (*X*^*2*^ = 15.54, df = 1, *P* = <0.0001). There was no correlation between the size of *T*. *moseri* males and females when found in heterosexual pairs (*P* = 0.5560, *r*^2^ = 0.0085, F = 0.3517, df = 42). Twenty-six percent of the ascidians harboring crabs of defined sex harbored two females (n = 15) and not a single ascidian harbored two males at the same time. Thirteen ascidians harbored a pair of crabs in which one crab was a juvenile: one male+juvenile and twelve female+juvenile pairs. Nineteen ascidians harbored three crabs: six trios comprising two males and one female, three trios comprising two females and one male, one trio comprising one male + one female + juvenile, six trios comprising three females, and three trios comprising two females and one juvenile (Fig 3). A single ascidian harbored sixteen crabs; one brooding female + 15 newly released crab instars. This ascidian was not included in our analysis of population distribution in *T*. *moseri*.

### Territoriality in *Tunicotheres moseri* adults

In the first experiment, in which we tested for territoriality in adult female crabs while exposing them simultaneously to a single unoccupied ascidian, no signs of aggression between crabs were observed. *Tunicotheres moseri* females did not employ a single agonistic behavior known to be associated with territoriality in other crustaceans [12, 39] or defined in the methods herein as agonistic behaviors. During the same experiment (n = 40), small crabs entered the host first on 16 occasions while larger crabs entered the host first on 24 occasions. These proportions were not statistically significant (*X*^*2*^ = 1.60, df = 1, *P* = 0.2059), indicating no influence of body size on host colonization by crabs.

**Fig 5:**
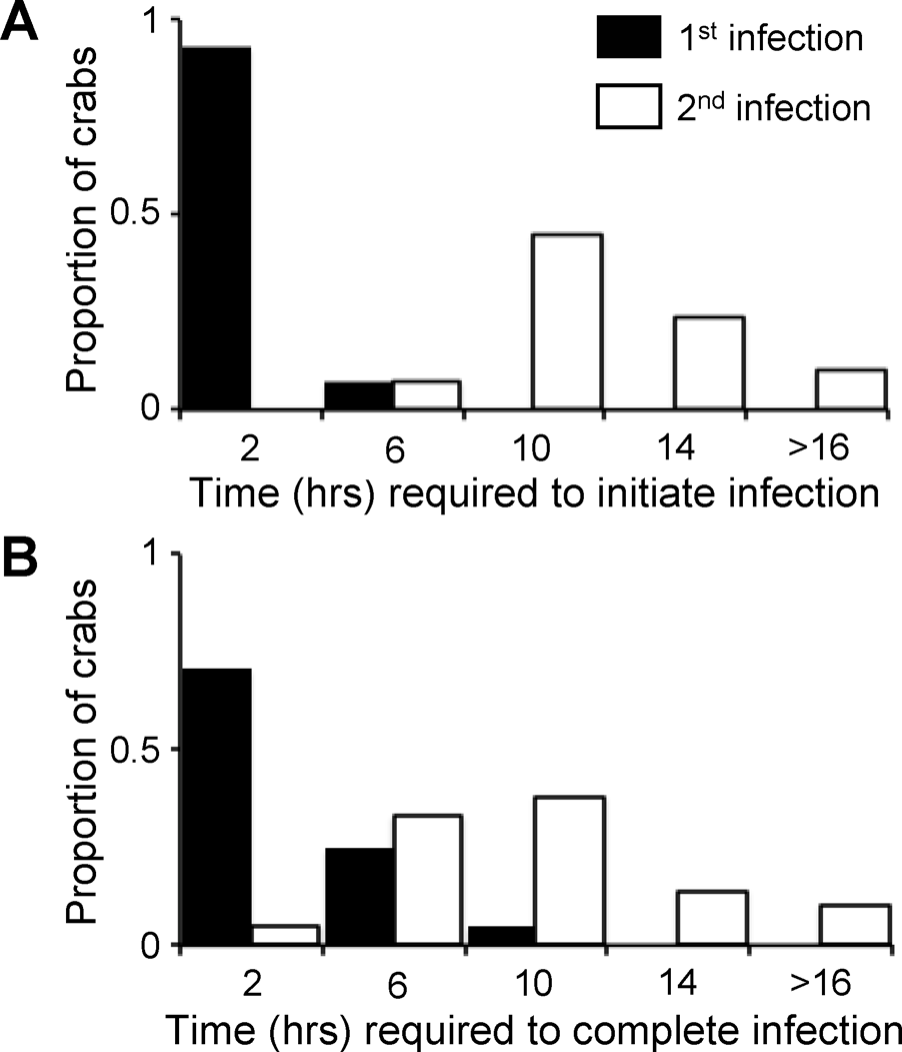
Time to initiate and complete colonization of uninfected and infected hosts. A comparison of the time required by searching *Tunicotheres moseri* adult crabs to (a) initiate and (b) complete colonization of ascidian hosts *Styela plicata* when crabs were offered a vacant (1^st^ infection) or occupied (2^nd^ infection) host.

Although no obvious agonistic behaviors between crabs were observed in the first experiment above, results from the second experiment suggest that resident crabs defend 'their' host individuals from intruder crabs. The time taken to initiate and complete an infection (mean ± SE) was significantly shorter when searching crabs were offered an uninfected compared to an occupied host (time to initiate infection: 3.88 ± 3.41 and 16.21 ± 4.53 h, respectively; Wald Chi-square test: *X*^*2*^ = 37.19, df = 1, *P* < 0.0001, time to complete infection: 2.78 ± 1.45 and 9.89 ± 3.25 h, respectively; Wald Chi-square test: *X*^*2*^ = 36.80, df = 1, *P* < 0.0001) (Fig 5). Also, experimental crabs invariably colonized host individuals that were not infected with a conspecific (85 out of 85, or 100%). By contrast, 90% of those crabs offered an infected host successfully colonized the target ascidian (38 out of 42, or 90%). The proportions above were statistically significant (Fisher’s exact test, df = 1, *P* = 0.011). We also found that smaller intruder crabs successfully colonized hosts (17 out of 21, or ~81%) almost as often as larger intruder crabs (21 out of 21, or 100%) indicating no effect of body size on colonization success (Fisher’s exact test, df = 1, *P* = 0.107).

Dissections of ascidians conducted after experiments 1 and 2 demonstrated that resident crabs were invariably located far from the siphon and near the bottom of the atrial chamber of their host ascidians when in the absence of intruder crabs. By contrast, resident crabs positioned themselves just inside the host atrial siphon opening (point of entrance to ascidians) when in the presence of an intruder crab (n = 4 and n = 5 crabs observed in experiments 1 and 2, respectively).

### Conflict avoidance in *Tunicothers moseri*

**Fig 6:**
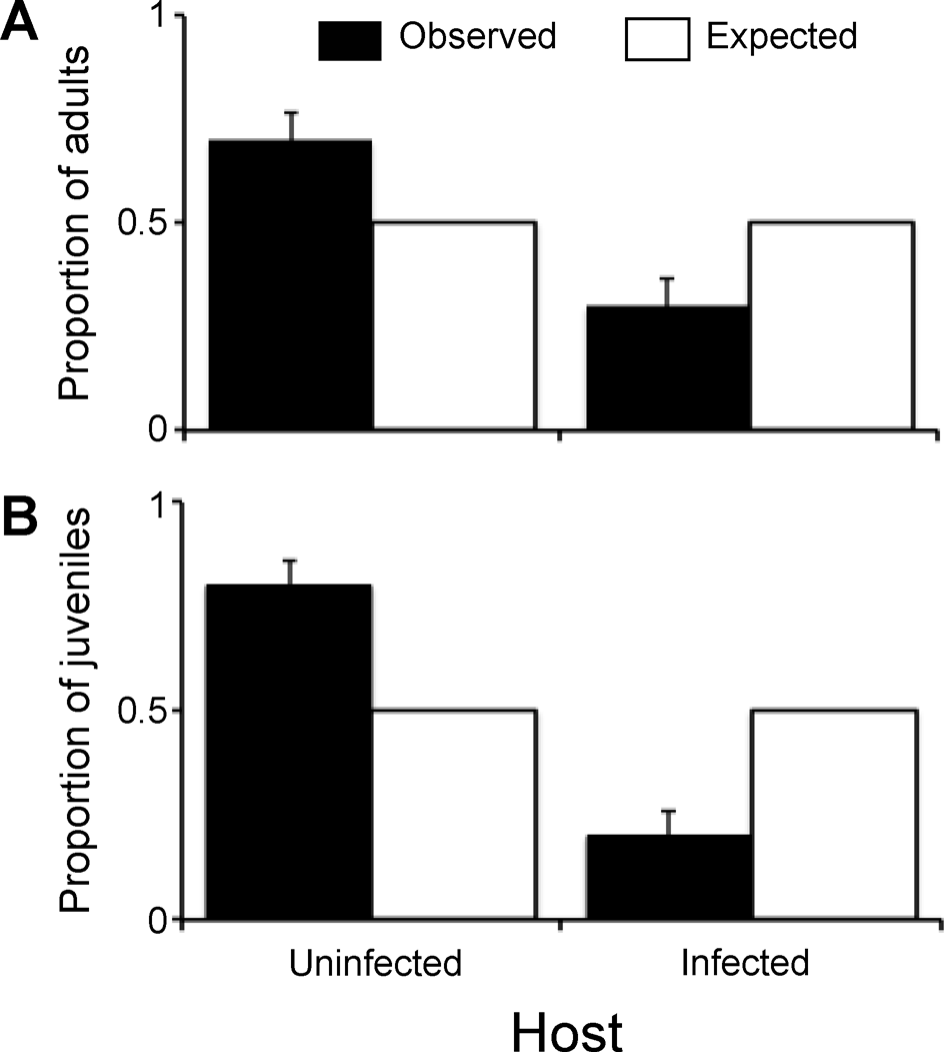
Preference by *Tunicotheres moseri* for uninfected and infected hosts. Proportion of (a) adult and (b) juvenile *Tunicotheres moseri* crabs actively avoiding previously infected *Styela plicata* hosts and preferring uninfected *S*. *plicata* hosts when offered both choices simultaneously. Error bars denote the standard error of the proportion of observed crabs choosing uninfected or infected hosts.

Adult females of *T*. *moseri* (n = 42) demonstrated a significant preference for uninfected hosts (n = 33) over infected ascidians (n = 8) (*X*^*2*^ = 15.24, df = 1, *P* < 0.0001) when offered both options simultaneously (Fig 6). Similarly, newly released instars (n = 31) chose uninfected hosts more often than expected (n = 23.5) by a binomial distribution (*X*^*2*^ = 4.79, df = 1, *P* = 0.0287) (Fig 6). These findings indicate that both adults and juveniles of *T*. *moseri* display conspecific avoidance.

## Discussion

Taking into account theoretical considerations [3], in this study, we predicted a solitary habit and territorial behavior in *T*. *moseri*. Our field observations mostly agree with the notion that this symbiotic crab lives solitarily in its ascidian host *S*. *plicata*. Over the study period, infected ascidians harbor a single symbiotic crab 71% of the time, and the frequency of crabs living solitarily in ascidians was significantly greater than that expected by chance alone in summer and fall (but not winter and spring, see below). The solitary habit herein reported for *T*. *moseri* agrees with previous observations of a southern Caribbean population of the same species (i.e., Venezuela–[24]) and with that reported for other symbiotic crustaceans inhabiting small and discrete hosts (e.g., *Allopetrolisthes spinifrons*: [3]; *Ascidonia flavomaclata*: [51]; *Calyptraeotheres garthi*: [52]).

*Tunicotheres moseri* primarily lives a solitary lifestyle, but we also observed seasonal fluctuations in the population distribution of this crab. Hosts harboring solitary crabs with a frequency greater than expected by chance were observed during summer and fall when host density was the highest. By contrast, the lowest occurrences of solitary crabs were reported during winter and spring seasons, when host densities were the lowest. At first glance, these findings seem to disagree with theoretical considerations [3] as we expect that low host abundance should reinforce territorial behaviors and the solitary habit of *T*. *moseri*. We believe that seasonal differences in the abundance of the crab at the study site might explain, at least partially, our results. Tampa Bay is a seasonal environment with low water temperatures from December to April compared to the rest of the year [53]. Recruitment of *T*. *moseri* to the benthic population often fails, the cause being unknown, during winter and spring seasons while dramatic population increases occur during summer and fall (LJ Ambrosio, unpublished data). The effect of seasonality on the mean abundance of *T*. *moseri* is also evidenced in our study by the low number of crabs collected during winter and spring compared to the other two seasons. Thus, although, absolute host abundance was greater during summer and fall than during winter and spring, many more crabs were present in the population during the warmer seasons of the year, likely resulting in greater host scarcity and competition among crabs for the relatively few unoccupied hosts (only ~21% and ~19% of ascidians were available [unoccupied] to crabs in summer and fall, respectively). Additional studies on the population dynamics of *T*. *moseri* in Tampa Bay are needed to understand the effects of host availability and crab density in determining the population distribution of this and other symbiotic species. As yet, our results suggest that *T*. *moseri* is primarily, a solitary species, in agreement with theoretical considerations.

### Does territoriality explain the solitary lifestyle in *Tunicotheres moseri*?

In this study, we also predicted that the solitary habit of *T*. *moseri* was explained by territorial behavior given the ecology and biology of its ascidian host [3]. Our experiments support, but only partially, this prediction. Although, no obvious agonistic displays were observed between crabs attempting to colonize a single uninfected host individual, intruder crabs took longer to initiate (~12.33 h longer) and complete (~7.11 h longer) an infection when offered an infected ascidian than when offered uninfected ascidians. Importantly, during the same experiment, dissections demonstrated that resident crabs positioned themselves just inside the host atrial siphon opening (point of entrance to ascidians by intruder crabs) when in the presence of an intruder. By contrast, resident crabs were located far from the siphon and near the bottom of the atrial chamber when in the absence of intruder crabs. We interpret these results as evidence of territoriality in *T*. *moseri*: resident conspecifics likely actively attempt to prevent colonization of 'their' host individuals by other (intruder) crabs and this behavior might explain why intruder crabs took longer to colonize occupied vs. unoccupied host individuals.

*Tunicotheres moseri* does monopolize ascidians and actively attempts to prevent intruder conspecifics from colonizing 'their' host individuals. However, the territorial behavior reported herein for *T*. *moseri* does not mirror the overt, sometimes fierce, physical contests documented for other symbiotic crustaceans competing for host individuals [12, 23, 33]. For instance, in *Allopetrolisthes spinifrons*, a porcelain crab symbiotic with sea anemones, crabs fiercely defend host individuals against conspecific intruders [23]. Agonistic encounters among adult porcelain crabs can and do result in serious injuries, including the complete ablation of massive chelipeds [23]. By contrast, in *T*. *moseri*, the observed behavior and position of resident crabs (near the opening of the ascidian atrial siphon) during our experiments suggests that these crabs, although actively blocking the invasion to their 'dwelling' by intruder crabs, do no fight fiercely for host individuals.

It is unclear why agonistic interactions between competing *T*. *moseri* crabs are not exaggerated and do not escalate into intense combat as commonly reported in other crustaceans, including symbiotic species [12, 23, 33]. In this endosymbiotic species, the frequency of host switching, and therefore, encounter rates among adult crabs might be rare, and thus, selection for overt behavioral displays and weaponry might be weak [54]. Furthermore, the evolutionary expression of exaggerated weaponry (e.g., massive chelipeds) might be constrained in *T*. *moseri* given that the atrial chamber of ascidians likely represents a fragile refuge for crabs. In support of this idea, *T*. *moseri* exhibits a smooth and spherical body form with small chelipeds and devoid of sharp projections [55]. Large well-ornamented (i.e., spines, tubercles, chelipeds) body structures might harm the internal soft tissue of ascidians, thus, constraining the evolution of weaponry in *T*. *moseri*. Although weaponry and agonistic behavior are poorly developed in *T*. *moseri*, our results suggest that territoriality plays, at least to some extent, a role in driving the solitary habit of this symbiotic crab.

*Tunicotheres moseri* actively prevent intruder conspecifics from colonizing 'their' host individuals. Nonetheless, territoriality in *T*. *moseri* is not completely effective; e.g., during our experiments, the proportion of intruder crabs completing host infection decreased from 100% to only 90% when attempting to colonize infected versus non-infected ascidians. Furthermore, our field surveys and data presented elsewhere [24] demonstrate female cohabitation in the field (though relatively rare). By contrast, in other symbiotic and territorial species, resident individuals almost invariably prevent invasion of 'their' host individuals by intruders and host cohabitation by members of the same sex is most rare (e.g., *Trapezia intermedia* and *Trapezia digitalis*: [12]; *Allopetrolisthes spinifrons*: [23]). It is unclear why territoriality is not a highly effective strategy in *T*. *moseri*. Resource contests between individuals are usually (but not always) decided by asymmetries (i.e. body size, weight, and energy reserves) that determine the ability of contestants to win a fight [56–57]. In *T*. *moseri*, we expected larger crabs to outcompete smaller conspecifics when invading host individuals given that body size is a good predictor of contest outcome in crustaceans [12, 41]. Nonetheless, our results demonstrated that smaller intruders gained access just as often as larger intruders when competing for host individuals. In the field, increased exposure to predation in intruders compared to residents might be affecting crab fighting motivation and this putative asymmetry in motivational state may result in increased competitive advantage in intruders compared to resident crabs, regardless of the presence of other asymmetries, i.e., body size [58–60]. We argue in favor of additional studies examining intrinsic and extrinsic individual traits that might be important in influencing motivation in *T*. *moseri* to understand those conditions relevant in resolving territorial conflicts over host resources in symbiotic crustaceans.

### Avoidance as a strategy to resolve conflict in *Tunicotheres moseri*?

*Tunicotheres moseri* exhibits a solitary lifestyle and agonistic interactions among adults plays a role in determining its solitary habit. However, our results suggest that territoriality alone seems inadequate to completely explain the solitary habit of this species. We observed that female adults took longer to infect a host individual when occupied than when unoccupied by a crab. This extra time required for infection likely implies considerable costs to intruder crabs [1]. Costs might include increased energy investment required to infect a host and increased exposure to predation outside of host individuals [61–62]. These putative costs are expected to favor (alternative) mechanisms other than territoriality to gain access to host individuals [63]. Accordingly, we suggested that the solitary habit of symbiotic species might, alternatively or additionally, be explained by conflict avoidance: the active avoidance of occupied hosts and preference for vacant hosts. The preference of adult females and newly released juveniles for uninfected over infected host individuals strongly support the notion above.

Our results confirm the ability of *T*. *moseri* adults to sense conspecifics within host individuals using tactile and/or chemical cues [34]. This ability likely diminishes direct (i.e., damage) and indirect (e.g., host search after displacement) costs associated with territorial disputes. Previous studies have demonstrated conflict-avoidance mechanisms in predator-prey systems [64–66] and during inter-and intraspecific competitive interactions [67–70]. For instance, the amphidromous shrimp *Atya lanipes* alters its activity level, habitat use, and subsequently, its population distribution when exposed to chemical and/or tactile cues from the predatory shrimp *Macrobrachium carcinus* [64]. Similarly, the parasitoid wasp *Leptopilina heterotoma* uses chemical cues to avoid larval host patches previously exploited by both con- and heterospecific competitors when depositing eggs [70]. Interestingly, the parasitoid example above represents a rare documentation of avoidance as a mechanism mediating competitive conflicts between conspecifics. To the best of our knowledge, the present study is the first with crustaceans to demonstrate a long distance mechanism (i.e., avoidance of used refuges) that diminishes conflict (and related costs) in adult individuals within the context of intra-specific competition for refuges. Whether or not conflict avoidance is a commonly employed strategy to mediate conspecific conflicts over resources remains a subject requiring additional research.

Adult females of *T*. *moseri* live solitarily, a habit explained by their territorial behavior and preferences for unoccupied host individuals. Our field data demonstrates that not only adult but also juvenile crabs live solitarily. Indeed, very few instances of juvenile-adult cohabitation were observed at our study site. According to our experiments, *T*. *moseri* juveniles avoided infected hosts and actively chose vacant hosts during initial host settlement. Altogether, this information suggests that conflict avoidance is relevant not only during adulthood but also during early ontogeny of *T*. *moseri*. Conflict avoidance might be particularly important for juveniles because early developmental stages often lack competitively advantageous traits (i.e. large body size or well developed chela), thus rendering them competitively inferior during contests with well-endowed adults [71–73]. Previous studies have documented heterospecific avoidance during settlement in a variety of invertebrate taxa and this avoidance strategy has additionally been shown to be relevant in influencing adult population distribution patterns [67, 74–75]. For instance, Grosberg [67] found that competitively inferior larvae would avoid settling near species that consistently outcompeted them later in life even if enough vacant substrate was available for settlement. As is indicated by the studies above and data presented herein, the solitary habit of *T*. *moseri* is probably established at settlement as a means of minimizing costly conflicts with adult conspecifics and is maintained throughout life by post settlement avoidance behaviors and to a lesser extent territoriality.

## Conclusion

Our study supports the prediction that symbiotic crustaceans inhabiting morphologically simple, relatively small and scarce hosts are territorial, a behavior that consequently explains their solitary lifestyle. Our results further suggest that territoriality alone is not an effective strategy to monopolize host individuals and that it is not the single mechanism explaining the solitary habit observed for *T*. *moseri*. Our experiments further demonstrate that *T*. *moseri* employs another important mechanism when colonizing host individuals, conflict avoidance behaviors, in which both adult and juvenile crabs avoid intra-specific conflict demonstrated by preference for unoccupied host individuals. Our results provide evidence for a relatively novel strategy of conflict resolution in resource-specialist (symbiotic) organisms. Whether or not behavioral avoidance is adaptive in other resource-specialist (symbiotic) organisms remains to be addressed.

## References

[1] Smith JM. The theory of games and evolution of animal conflicts. J Theor Biol. 1974;47:209–221. 4459582 10.1016/0022-5193(74)90110-6

[2] Grant JWA, Guha RT. Spatial clumping of food increases its monopolization and defense by convict cichlids, *Cichlosoma nigrofasciatum*. Behav Ecol. 1993;4(4):293–296.

[3] Baeza JA, Thiel M. Predicting territorial behavior in symbiotic crabs using host characteristics: a comparative study and proposal of a model. Mar Biol. 2003;142:93–100.

[4] Brown JL. The evolution of diversity in avian territorial systems. Wilson Bull. 1964;76:160–169.

[5] Grant JWA. Whether or not to defend? The influence of resource distribution. Mar Freshw Behav Physiol. 1993;22:137–153.

[6] Emlen ST, Oring LW. Ecology, sexual selection and the evolution of mating systems. Science. 1977;197:215–223. 327542 10.1126/science.327542

[7] De Bruyn C, Rigaud T, David B, De Ridder C. Nature and consequences of the symbiotic relationship between the crab *Dissodactylus primitivus* and its echinoid host *Meoma ventricosa*. Mar Ecol-Prog Ser. 2009;375:173–183.

[8] Grand TC, Grant JWA. Spatial predictability of food influences its monopolization and defense by juvenile convict cichlids. Anim Behav. 1994;47(1):91–100

[9] Brooks WR. Hermit crabs alter anemone placement patterns for shell balance and reduced predation. J Exp Mar Biol Ecol. 1989 11 3;132(2):109–121.

[10] Langkilde T, Shine R. Competing for crevices: interspecific conflict influences retreat-site selection in montane lizard. Oecologia. 2004;140(4):684–691. 15252729 10.1007/s00442-004-1640-1

[11] Hack MA. The energetic cost of fighting in the house cricket, *Acheta domesticus* L. Behav Ecol. 1997;8(1):28–36.

[12] Huber ME. Aggressive behavior of *Trapezia intermedia* Miers and *T*. *digitalis* Latreille (Brachyura: Xanthidae). J Crustacean Biol. 1987;7:238–248.

[13] Palombit RA. Lethal territorial aggression in a white-handed gibbon. Am J Primatol. 1993;31(4):311–318. 10.1002/ajp.1350310407 31936991

[14] Brante A, Viard MF. Non-random sibling cannibalism in the marine gastropod *Crepidula coquimbensis*. PLOS ONE. 2013 6 21;8(6):e67050 23805291 10.1371/journal.pone.0067050 PMC3689673

[15] Chapman MR, Kramer DL. Guarded resources: the effect of intruder number on the tactics and success of defenders and intruders. Anim Behav. 1996;52:83–94.

[16] Both C, Visser ME. Density dependence, territoriality, and divisibility of resources: from optimality models to population processes. Am Nat. 2003;161(2):326–336. 12675376 10.1086/346098

[17] Duffy JE. *Synalpheus regalis*, new species, a sponge-dwelling shrimp from the Belize Barrier Reef with comments on host specificity in *Synalpheus*. J Crustacean Biol. 1996;16(3):564–573.

[18] Abele LG. Comparative species richness in fluctuating and constant environments: coral-associated decapod crustaceans. Science. 1976 4 30; 192(4238):461–463. 17731083 10.1126/science.192.4238.461

[19] Calado R, Dionísio G, Dinis MT. Decapod crustaceans associated with the snakelock anemone *Anemonia sulcata*. Living there or just passing by? Sci Mar. 2007;7(2):287–292.

[20] Baeza JA. Social monogamy in the shrimp *Pontonia margarita*, a symbiont of *Pinctada mazatlantica*, off the Pacific coast of Panama. Mar Biol. 2008 153:387–395.

[21] Wells HW, Wells MJ. Observations on *Pinnaxodes floriden*sis, a new species of pinnotherid crustacean commensal in holothurians. Bull Mar Sci. 1961;11:267–279.

[22] Diesel R. Male-female association in the spider crab *Inachus phalangium*: the influence of female reproductive stage and size. J Crustacean Biol. 1988;8:63–69.

[23] Baeza JA, Stotz W, Thiel M. Agonistic behaviour and development of territoriality during ontogeny of the sea anemone dwelling crab *Allopetrolisthes spinifrons* (H. Milne Edwards, 1987) (Decapoda: Anomura: Porcellanidae). Mar Freshw Behav Phy. 2002;35(40):189–202.

[24] Hernández JE, Bolaños JA, Palazón JL, Hernández G, Lira C, Baeza JA. The enigmatic life history of the symbiotic crab *Tunicotheres moseri* (Crustacea, Brachyura, Pinnotheridae): implications for its mating system and population structure. Biol Bull. 2012;223:278–290. 23264474 10.1086/BBLv223n3p278

[25] Telford M. Echinoderm spine structure, feeding and host relationships of four species of *Dissodactylus* (Brachyura: Pinnotheridae). B Mar Sci. 1982;32:584–594.

[26] Henkel TP, Pawlik JR. Habitat use by sponge-dwelling brittlestars. Mar Biol. 2005;146:301–313.

[27] Stebbins TD. Population dynamics and reproductive biology of the commensal isopod *Colidotea rostrata* (Crustacea: Isopoda: Idoteidae). Mar Biol 1989;101:329–337.

[28] Thiel M, Baeza JA. Factors affecting the social behaviour of crustaceans living symbiotically with other marine invertebrates: a modeling approach. Symbiosis. 2001;30:163–190.

[29] Thiel M, Zander A, Baeza JA. Movements of the symbiotic crab *Liopetrolisthes mitra* between its host sea urchin *Tetropygus niger*. B Mar Sci. 2003;72:89–101.

[30] Thiel M, Zander A, Valdivia N, Baeza JA, Rueffler C. Host fidelity of a symbiotic porcellanid crab: the importance of host characteristics. J Zool. 2003;261:353–362.

[31] Baez P, Martinez C. Desove y fecundidad de *Pinnaxodes chilensis* (H. Milne Edwards, 1837) (Crustacea, Decapoda, Brachyura, Pinnotheridae). Ann Mus Hist Nat Valparaiso so 1976;9:45–60. Spanish.

[32] Duffy JE. Host use patterns and demography in a guild of tropical sponge-dwelling shrimps. Mar Ecol-Prog Ser. 1992;90:127–138.

[33] Huber ME, Coles SL. Resource utilization and competition among the five Hawaiian species of *Trapezia* (Crustacea,Brachyura). Mar Ecol-Prog Ser. 1986 4 24;30:21–31.

[34] Ambrosio LJ, Brooks WR. Recognition and use of ascidian hosts, and mate acquisition by the symbiotic pea crab *Tunicotheres moseri* (Rathbun 1918): the role of chemical, visual and tactile cues. Symbiosis. 2011;53:53–61.

[35] Bolaños J, Cuesta J, Hernández G, Hernández J, Felder DL. Abbreviated larval development of *Tunicotheres moseri* (Rathbun, 1918) (Decapoda: Pinnotheridae), a rare case of parental care among brachyuran crabs. Sci Mar. 2004;68:373–384.

[36] Stoner AW, Livingston RJ. Ontogenetic patterns in diet and feeding morphology in sympatric sparid fishes from seagrass meadows. Copeia. 1984 2 23;1984(1):174–187.

[37] Harrigan P, Zieman JC, Macko SA. The base of nutritional support for the gray snapper (*Lutjanus griseus*): an evaluation based on a combined stomach content and stable isotope analysis. B Mar Sci. 1989;44(1):65–77.

[38] Elliot JM. Some methods for the statistical analysis of samples of benthic invertebrates Scientific publications 25 Freshwater Biological Association; 1971.

[39] Wright HO. Visual displays in brachyuran crabs: field and laboratory studies. Am Zool. 1968;8:655–665.

[40] Caldwell RL, Dingle J. The influence of size differential on agonistic encounters in the mantis shrimp *Gonodactylus viridis*. Behaviour. 1979;69:255–264.

[41] Adams ES, Caldwell RL. Deceptive communication in asymmetric fights of the stomatopod crustacean *Gonodactylus bredini*. Anim Behav. 1990;39:706–716.

[42] Ranta E, Lindström K. Power to hold sheltering burrows by juveniles of the signal crayfish, *Pascifastacus leniusculus*. Ethology. 1992;92(3):217–226.

[43] Jennions MD, Backwell PRY. Residency and size affect fight duration and outcome in the fiddler crab *Uca annulipes*. Biol J Linn Soc. 1996;57:293–306.

[44] Zar JH. Biostatistical analysis, 5^th^ edition. New York; 2009.

[45] Allison PD. Survival analysis using the SAS: a practical guide Cary: SAS Institute; 1995.

[46] SAS Institute Inc. JMP 12. Cary, NC SAS Institute; 2015.

[47] McDonald JH. Handbook of Biological Statistics, 3rd ed. Baltimore, Maryland Sparky House Publishing; 2014.

[48] Goldstien JS, Butler MJ. Behavioral enhancement of onshore transport by postlarval Caribbean spiny lobster (*Panulirus argus*). Limnol Oceanogr. 2009;54(4):1669–1678.

[49] Anderson JA, Epifanio CE. Responseof the Asian shore crab *Hemigrapsus sanguineus* to metamorphic cues under natural field conditions. J Exp Mar Biol Ecol. 2010;384:87–90.

[50] Elbourne PD, Clare AS, Ecological relevance of a conspecific, waterborne settlement cue in *Balanus amphitrite* (Cirripedia). J Exp Mar Biol Ecol. 2010;392:99–106.

[51] Baeza JA, Díaz-Valdés M. The symbiotic shrimp *Ascidonia flavomaculata* lives solitarily in the tunicate *Ascidia mentula*: implications for its mating system. Invertebr Biol. 2011;130(4):351–361.

[52] Ocampo EH, Nuñez JD, Cledón M, Baeza JA. Host specific reproductive benefits, host selection behavior and host use patterns of the pinnotherid crab *Calyptraeotheres garthi*. J Exp Mar Biol Ecol. 2012;429(1):36–46.

[53] Gandy R, Schott EJ, Crowley C, Leone EH. Temperature correlates with annual changes in *Hematodinium perezi* prevalence in blue crab *Callinectes sapidus* in Florida, USA. Dis Aquat Organ. 2015;113(3):235–243. doi: 10.3354/dao02841 25850401 10.3354/dao02841

[54] Baeza JA, Asorey CM. Testing the role of male-male competition in the evolution of sexual dimorphism: a comparison between two species of porcelain crabs. Biol J Linn Soc. 2012;105(3):548–558.

[55] Rathbun MJ. The grapsoid crabs of America. US Nat Mus Bull. 1918;97:1–461.

[56] Arnott G, Elwood RW. Information gathering and decision making about resource value in animal contest. Anim Behav. 2008;76:529–542.

[57] Arnott G, Elwood RW. Assessment of fighting ability in animal contests. Anim Behav. 2009;77:991–1004.

[58] Crowley PH, Gillett S, Lawton JH. Contests between larval damselflies: empirical steps toward a better ESS model. Anim Behav. 1988;36:1496–1510.

[59] Brown WD, Smith AT, Moskalik B, Gabriel J. Aggressive contests in house crickets: size, motivation and the information content of aggressive songs. Anim Behav. 2006;72:225–233.

[60] Brown WD, Chimenti AJ, Siebert JR. The payoff of fighting in house crickets: motivational asymmetry increases male aggression and mating success. Ethology. 2007;113:457–465.

[61] Bach CE, Herrnkind WF. Effects of predation pressure on the mutualistic interaction between the hermit crab *Pagurus pollicaris* Say, 1817, and the sea anemone *Calliactis tricolor* (Lesueur, 1817). Crustaceana. 1980;38(1):104–108.

[62] Brooks WR, Mariscal RN. Protection of the hermit crab *Pagurus pollicaris* Say from predators by hydroid-colonized shells. J Exp Mar Biol Ecol. 1985;87:111–118.

[63] Payne RJH, Pagel M. Escalation and time costs in displays of endurance. J Theor Biol. 1996;183:185–193.

[64] Crowl TA, Covich AP. Response of a freashwater shrimp to chemical and tactile stimuli fom a large decapod predator. J N Am Benthol Soc. 1994;13(2):291–298.

[65] Turner AM, Montgomery SL. Spatial and temporal scales of predator avoidance: experiments with fish and snails. Ecology. 2003;84(3):616–622.

[66] Zamzow JP, Amsler CD, McClintock JB, Baker BJ. Habitat choice and predator avoidance by Antarctic amphipods: the role of algal chemistry and morphology. Mar Ecol-Prog Ser. 2010 2 11;400:155–163.

[67] Grosberg RK. Competitive ability influences habitat choice in marine invertebrates. Nature. 1981;290:700–702.

[68] Sweatman H. Field evidence that settling coral reef fish larvae detect resident fishes using dissolved chemical cues. J Exp Mar Biol Ecol. 1988;124:163–174.

[69] Janssen A, Alphen JJM, Sabelis MW, Bakker K. Specificity of odour mediated avoidance of competition in *Drosophila* parasitoids. Behav Ecol. Sociobiol. 1995;36:229–235.

[70] Weiss I, Rössler T, Hofferberth J, Bummer M, Ruther J, Stökl J. A nonspecific defensive compound evolves into a competition avoidance cue and a female sex pheromone. Nat Commun. 2013 11 15;4:2767 doi: 10.1038/ncomms3767 24231727 10.1038/ncomms3767 PMC3868268

[71] Franke HD, Janke M. Mechanisms and consequences of intra- and interspecific interference competition in *Idotea baltica* (Pallas) and *Idotea emarginata* (Fabricius) (Crustacea: Isopoda): A laboratory study of possible proximate causes of habitat segregation. J Exp Mar Biol Ecol. 1998;227:1–21.

[72] Figler MH, Blank GS, Peeke HVS. Shelter competition between resident male red swamp crayfish *Procambarus clarkii* (Girard) and conspecific intruders varying by sex and reproductive status. Mar Freshw Behav Phy. 2005;38(4):237–248.

[73] Kobak J, Jermacz Ł, Dzierżyńska-Białończyk A. Substratum preference of the invasive killer shrimp *Dikerogammarus villosus*. J Zool. 2015;297(1):66–76.

[74] Young CM, Chia FS. Laboratory evidence for delay of larval settlement in response to a dominant competitor. Int J Inver Rep. 1981;3:221–226.

[75] Petersen JH. Larval settlement behaviour in competing species: *Mytilus californianus* Conrad and *M*. *edulis*. L. J Exp Mar Biol Ecol. 1984;82:147–159.

